# What Did They Mean by That? Young Adults' Interpretations of 105 Common Emojis

**DOI:** 10.3389/fpsyg.2021.655297

**Published:** 2021-05-31

**Authors:** Christopher A. Was, Phillip Hamrick

**Affiliations:** Kent State University, Kent, OH, United States

**Keywords:** emoji, emoji analysis, texting, emoji interpretation, emoji usage

## Introduction

Emojis are a form of ideograms, consisting of icons intended to represent facial expressions, emotions, objects, or other symbols, most commonly used in technologies such as smartphones, tablets, and computers. Although the smiley face (a commonly used emoji) first appeared in the 1960s (Bai et al., 2019), emoji use has recently become ubiquitous, with more than six billion being sent daily (Evans, 2017). Despite being discounted by some as a degraded form of communication that is ruining language (e.g., Jones, 2015), there is substantial linguistic evidence that emoji are not a threat to natural language, but, rather, they are a useful augmentation of natural language that enhance its communicative capacities (Evans, 2017). This is particularly true because the types of written communication found in text messages, emails, Tweets, and other such technologies are often prone to misinterpretation without paralinguistic cues (e.g., gesture, intonation, facial expressions). Emojis can function as paralinguistic information (Tantawi and Rosson, 2019), and they can help disambiguate alternative interpretations, convey emotion, sarcasm, and other kinds of information normally only available in spoken face-to-face communication (Holtgraves and Robinson, 2020). Indeed, emoji are so powerful in their communicative conveyance that an entirely emoji-based message was responsible for a teenager's alleged terrorist threat in New York in 2015 (Evans, 2017). Thus, emojis are a popular and robust form of meaning-making.

Emojis have also proven to be effective in studying cognitive phenomena without the influence of language. For example, Marengo et al. (2017) asked participants to respond to a brief Big Five Personality inventory and to 91 emojis drawn from the Apple Color Emoji fontset. Findings suggest that of the 91 emojis presented participant responses to 36 were correlated with their responses to three of the five personality traits. The responses to emojis were most related to emotions and affective processing. These researchers also empirically developed an emoji based instrument to assess depressive symptoms (Marengo et al., 2019). Marengo et al. (2019) conducted two studies to develop the emoji-based depression assessment. In the first study they asked young adults to indicate if each of the 36 emojis presented represented a way they felt during much of the past week. They also asked participants to complete a 10-item depression inventory. The association between the emojis and the depression measure items were calculated and the emoji with the 10 strongest associations with depression inventory items were tested for convergent validity and regression analyses allowed for accurate detection of depressive symptoms using the 10-item emoji scale.

The results of the studies by Marengo and colleagues demonstrate the utility of emojis beyond language, with opportunities to create and study language-free measures of various cognitive phenomena, including personality, depressive symptoms, and perhaps other individual differences. One possible way to increase the validity and robustness of such findings would be to use established norms regarding the interpretation of the emojis, thus allowing for experimental hypothesis testing regarding the relationships between emoji interpretation and other psychological constructs.

Despite their ubiquity and communicative power, the cognitive mechanisms supporting emoji comprehension and use remain elusive, although some research is starting to shed light on their role in semantic processing. For example, Weissman and Tanner (2018) examined whether emoji could induce language-like semantic processing. Event-related potentials were collected while participants read sentences like “The cake she made was terrible.” These sentences were followed by emoji that matched (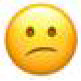), mismatched (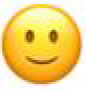), or that indicated irony or sarcasm (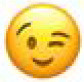). Relative to those control trials, irony and sarcasm emojis elicited P600 and P200 event-related potentials, similar to the potentials elicited by purely verbal irony, suggesting that emoji and language might be processed similarly, at least in the case of irony and sarcasm. Emojis appear to also enhance regular language processing. Chatzichristos et al. (2020) found that emojis (compared to pseudoword controls) trigger more complex processing of the words they are paired with. Emojis also appear to facilitate meaning comprehension when the meaning of an utterance is indirect (Holtgraves and Robinson, 2020). Not only can emojis facilitate comprehension of language, but they also can aid in disambiguating other emojis. For example, adding a wink emoji to a message with food emojis that are not associated with sexual euphemisms can lead those same food emojis to be interpreted in a sexual way (Weissman, 2019). These findings make all the more sense in light of a recent study by Gantiva et al. (2020), who found that emoji faces elicited similar neural responses to human faces, suggesting again that emojis can provide paralinguistic information that is typically available in face-to-face, but not in text-based, communication. Emoji interpretation has also been studied without surrounding linguistic context. For example, Miller et al. (2017) examined definitions and sentiment ratings of ambiguous emojis, finding that emoji interpretation in context was not significantly less ambiguous than when they were interpreted standalone.

There are at least two important caveats in much of the available research on how emojis are interpreted. First, the number of unique emojis used as stimuli has been restricted to a small pool of possible stimuli. Second, the “meanings” of the emojis used in these studies have often been assigned based on the researcher's intuition, rather than on the basis of data from norming studies. This may be particularly problematic in the case of emojis, which are very popular among the young adults who generally serve as participants in research. Given that research has shown differences between older and younger adults in emoji use (Hsiao and Hsieh, 2014) and interpretation (Gallud et al., 2018), it seems important for researchers to have a better sense of how young adults understand and interpret emojis before they begin designing their stimuli.

Some researchers have attempted to gather such normative data. For example, Novak et al. (2015) used natural language processing tools to develop a publicly available sentiment association inventory for emojis based on the distributions of words that co-occurred with emojis in the social media platform Twitter. However, it is not clear how well such an inventory maps onto actual human ratings. Human ratings of emojis have been elicited in previous research (e.g., Miller et al., 2017), but these data have not been made publicly available to our knowledge.

One set of normative data regarding emojis available to the public was produced by Rodrigues et al. (2018). Rodrigues et al. presented 258 stimuli (85 emoticons and 153 emojis) from the Lisbon Emoji and Emoticon Database to more than 500 Portuguese participants. Each participant rated 20 random stimuli on seven dimensions (e.g., valence, concreteness, meaningfulness). The results of the analysis indicated that emojis were found to have more aesthetic appeal and to be more meaningful than emoticons. Germaine to the current study, results of their meaning analysis suggest that intending meaning of the use of an emoji and the interpretation of the emoji are not always perfectly correlated. We believe that the Rodrigues et al. norms are useful for researchers as a set of norms however, we hope to provide a more comprehensive understanding of the way in which common emojis are interpreted. Our study also contributes to the literature as we conducted our study with English speaking participant using English responses, whereas the participants in the Rodrigues et al. (2018) were native Portuguese speakers.

Given the growing interest in understanding the effects of emojis in semantic processing and given that there appears to be little or no publicly available data on how emojis are interpreted by the kinds of young adults that commonly participate in research, the aim of this study was to obtain data on how emojis are interpreted by young adults and to make those data and some code for how to parse them and use them for future research publicly available. These emoji interpretations could be, for example, (i) used in developing controlled experiments, (ii) compared with their meaning in context, (iii) can act as a baseline for computational accounts of emoji in meaning, and so on. To that end, this study provides a data set of 105 emoji from 129 participants. Sentiment (emotional valence) data and (multi)word associations for each emoji were elicited. Subject-level data on their use of emojis and text messaging behaviors was also elicited. The data are publicly available in their raw form and sample code for data manipulation, cleaning, and analyses are also available *via* the Open Science Framework at https://osf.io/za65c/.

## Methods

### Participants

One hundred twenty-nine undergraduate psychology students at a large Midwestern University participated in the study for course credit. Eighty-eight participants identified as female and 41 as male. Participants were recruited from the university's Psychology Participant Pool *via* an online recruiting system. The age of participants ranged from 18 to 60 (*M* = 20, *SD* = 4.62). The university's Institutional Review Board approved the design of the study. Written consent was not collected as participants were ensured of the anonymity of the study and no identifying information was collected.

### Materials

105 emojis were selected from the Apple® iPhone list of emojis as presented on the Emojipedia website (Emojis were selected based on their appearance in order in Apple's list of emojis (the emojis are viewable in the Open Science Framework https://osf.io/za65c/). The aim of the study was to present usable public data from a homogeneous pool of stimuli (e.g., controlled for size, coloration, etc.) from the most popular platform available. In this case, we chose emojis from Apple OS given available data on its popularity among college-aged people (e.g., among college-aged people in the USA, the Apple iPhone accounts from the majority of smartphone brand use, at ~40% of users according to Statista).

That said, despite our goal of homogeneity of the pool of emojis, although we chose emojis from the Apple OS, the basic emoji symbols are virtually the same on iOS and Android as approved by the Unicode Consortium. The Apple and Google designers do create different looks for each icon and the names of the emojis are typically not representative of the emotions evoked, but are descriptive of the emoji itself (e.g., face with rolling eyes). In cases of emojis representing hand gestures, we randomly chose one example from the multiple skin tone representations. The study was programmed in E-Prime 3.0® and delivered to participants *via* the participant recruitment system as an E-Prime Go executable file.

### Procedures

After scheduling participation in the study *via* the online participant recruitment system, participants downloaded the E-Prime Go file to their personal computer. Participants were informed that the study was designed to acquire individuals' interpretation of common emojis. The instructions informed participants that they would be shown emojis one at a time on their computer screen and asked to describe what the emoji was meant to represent and rate how strongly positive or negative the emotion related to the emoji occurred to them.

Following the instructions, the participants were asked how often they send text messages. Participants replied using the following scale:

1 = 20 or more times per day.
2 = 10 or more times per day.
3 = 2 or more times per day.
4 = I rarely text.
5 = I never text.

They were also asked to indicate how often they use emojis. They replied using the following scale:

1 = I include an emoji in almost every message I send.
2 = I use emojis in some messages.
3 = I rarely use emojis in my messages.
4 = I never use emojis in my messages.

The 105 emojis appeared one at a time at the center of the computer screen. Above each emoji was the prompt “What word or phrase comes to mind with this emoji?” Participants typed in their response in a window appearing below the emoji and used the Enter key to complete their response. Following their response, the emoji remained in the center of the screen and the prompt “How strong is the emotion related to this emoji?” appeared above the emoji and a 5-point Likert-type scale ranging from 1-very negative to 5-very positive appeared below the emoji. Participants recorded their response using the numbers on their keyboard and the next emoji appeared. This procedure continued until participants responded to all 105 emojis. Participants' data was automatically saved to the desktop of their computer. Participants emailed the zip file containing the data to an email dedicated for data collection.

## Results

In this section, we briefly describe some of the statistical characteristics of participants' responses, and we give some examples of the kinds of analyses that can be conducted with these data.

### General Characteristics of the Data

**Figure 1:**
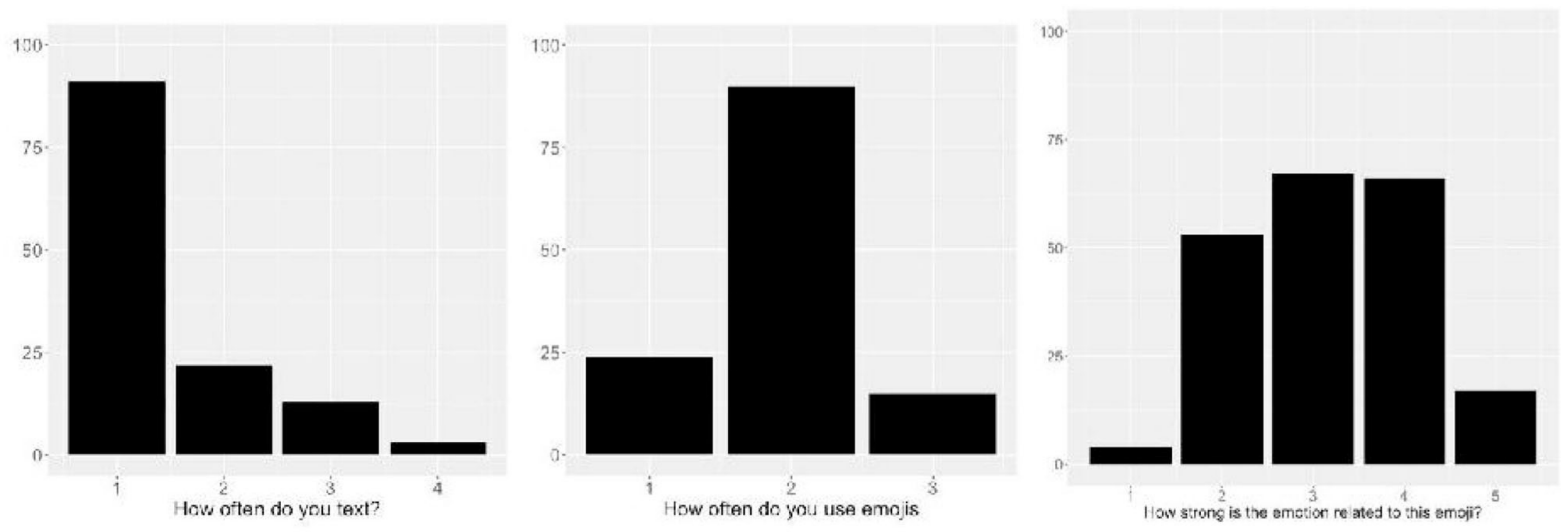
Histograms of text use **(A)**, emoji use **(B)**, and emotional valence **(C)**.

Table 1 presents text use, emoji use, emotional valence of emoji, and number of words produced per emoji by self-reported sex. As expected, there was little variation among the participants in their text and emoji use. Participants generally reported very frequent use of text messaging (*M* = 1.44, *SD* = 0.77, *SE* = 0.07, range = 1–4). Similarly, participants reported frequently using emojis (*M* = 1.93, *SD* = 0.55, *SE* = 0.05, range = 1–3). No participants reported that they never text or never use emojis. The median emotional valence rating on a scale from 1 (very negative) to 5 (very positive) was computed for each emoji. The median of these values was 3, with some degree of variability across all emojis (*SD* = 0.95, *SE* = 0.09), and all possible valence values were used (range 1–5) (See Figure 1).

**Table 1:** Text use, emoji use, emotional valence of emoji, and number of words produced per emoji by self-reported sex.

	**Females**	**Males**
Text use	1.40 (0.75)	1.54 (0.81)
Emoji use	1.82 (0.56)	2.17 (0.44)
Emotional valence	3.20 (0.98)	3.17 (0.97)
Number of words per emoji	1.56 (0.32)	1.39 (0.27)

**Figure 2:**
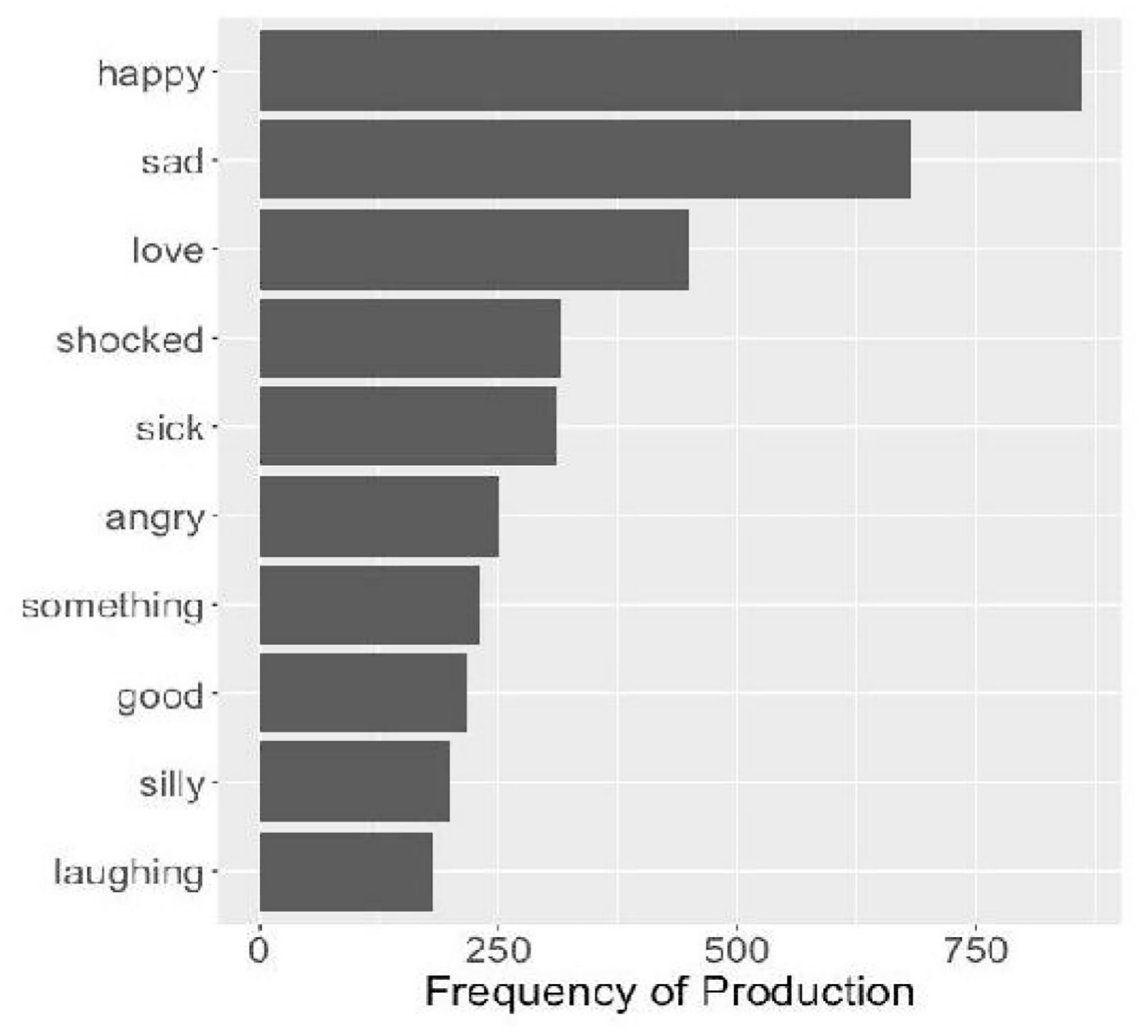
The 20 most frequently produced individual words overall.

Participants produced an average of 1.51 individual words (SD = 0.30, SE = 0.03, min = 1.22, max = 3.03) for each emoji, sometimes as strings of single words, sometimes expressing a more complex interpretation (e.g., “crying laughing,” “mind blown”). Figure 2 presents the 20 most produced words overall. Importantly, the raw data made available include participants' original responses, and researchers wishing to use these data can either work with the individual words, participants' multi-word responses, or both. Code for converting the data to a one-word-per-row are included in the R code also made available in this data report.

### Other Possible Analyses

**Figure 3:**
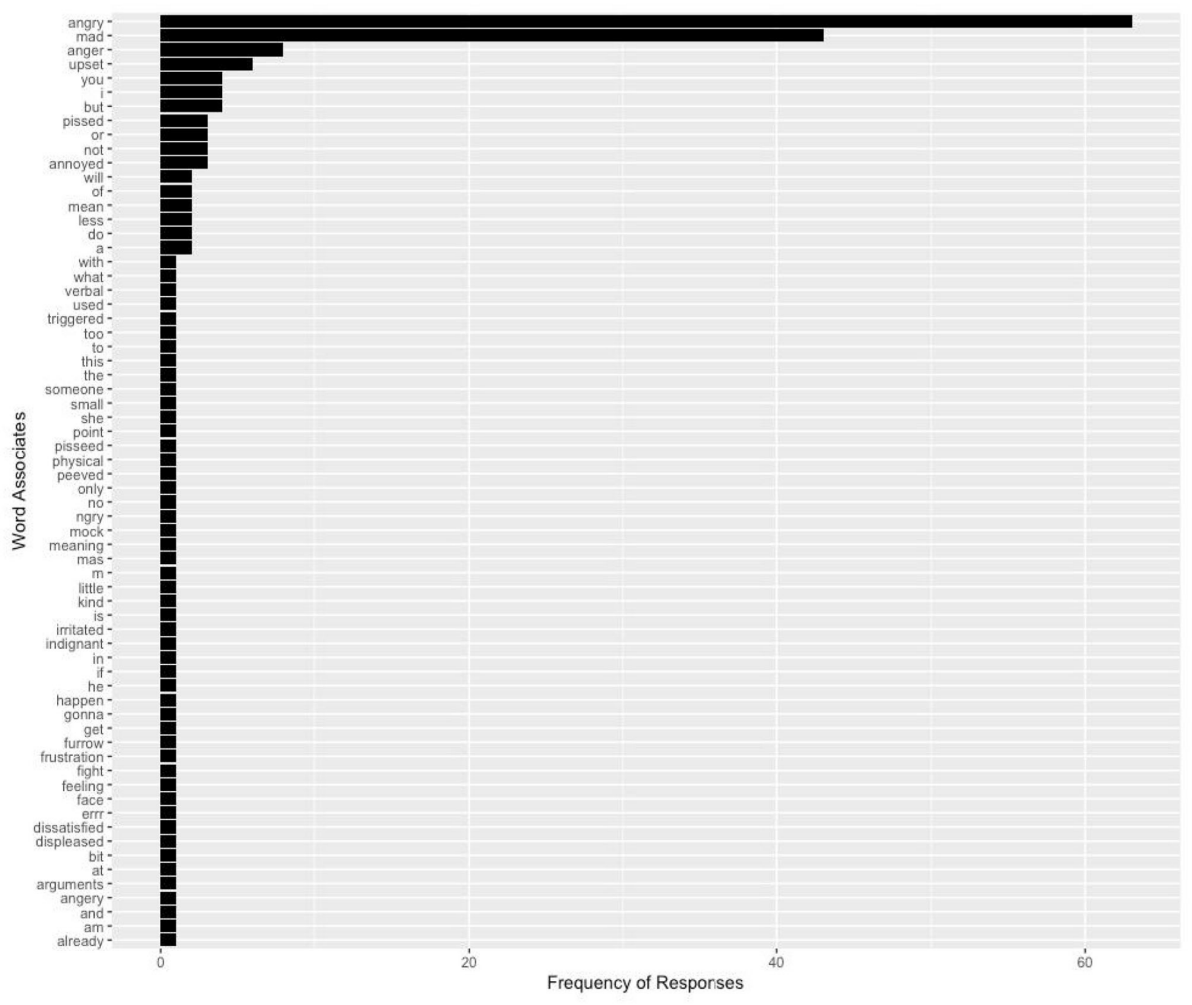
Word associations produced in response to 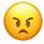 (angry face). Note that the word responses here have not been filtered to remove stopwords (e.g., “to”) nor have they been modified (e.g., to correct spelling errors).

The R code included with this data set also provides code that allows researchers to merge the emoji data with other data frames. Word-level data (e.g., word frequency, contextual diversity, orthographic neighborhood density, etc.) from existing databases (e.g., the English Lexicon Project, MRC Psycholinguistic Database, etc.) can be easily imported and entered into subsequent analyses. A potentially fruitful avenue for future research on how emojis are interpreted and how they influence meaning comprehension could be to study psycholinguistic properties of the words they have elicited in this dataset. Each emoji in our data set can produce a variety of word associations (e.g., see Figure 3), and those could be explored along several lines. For example, do certain emoji elicit more concrete or abstract word associations? It also may prove interesting to see the range of different emoji associated with a single word associate (Table 2).

**Table 2:** Emoji stimuli that elicited the word associate “happy” as a response at least 10 times across subjects (this is an arbitrary boundary used for simplicity of presentation).

**Emoji**	**Frequency with which “happy” is produced as a response**
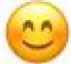	141
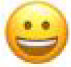	88
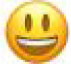	86
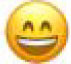	85
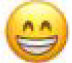	75
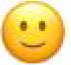	71
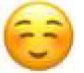	49
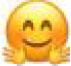	34
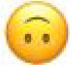	27
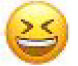	22
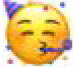	21
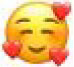	19
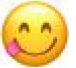	14
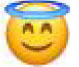	12
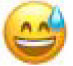	11
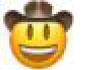	10
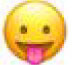	10
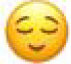	10

We also believe that another illuminating avenue for research will be to examine whether the semantic similarity of any two given emojis is comparable when using purely distributional information (e.g., Novak et al., 2015) compared with the emoji-(multi)word associations produced by our participants. In order to do so, one must convert the qualitative (multi)word responses into quantitative data. One way to do that is by using word vectors, which themselves are also learned distributionally. In the supplemental R code, we use pre-trained word vectors^1^ from a prominent distributional model of semantic memory, the Bound Encoding of the Aggregate Language Environment model (BEAGLE; Jones and Mewhort, 2007). The BEAGLE model produces word vectors that reflect both word context (e.g., semantic co-occurrence) and word order (e.g., syntax). These word vectors can then be compared (e.g., by computing their cosine) to determine their similarity, and by extension, the similarity of the words they represent. When a given emoji elicits one or more words, these vectors can be averaged to produce a composite (e.g., a “prototype” meaning for the emoji) or even compared against one another to determine how much internal consistency there is in the meaning of a given emoji.

In the accompanying R code, we include the necessary code to import these vectors (vectors from other distributional models could also be used) along with code for computing cosine similarity between all average emoji vectors (we used a one-word-per-row format to do this, but it is possible to use participants' multi-word responses). These can be examined at a large scale, used for clustering emoji into groups, or for simply computing meaning similarity between individual pairs of emoji. For example, the cosine similarity between 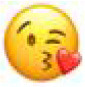 and 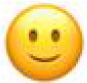, 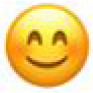 is very high (cos = 0.92), but 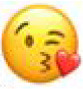 is much less similar to 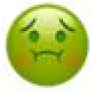 (cos = 0.49).

We acknowledge that there other publicly available data sets and analyses of emojis (e.g., Barbieri et al., 2016a,b), but our data set introduces publicly available human ratings that could act as human benchmarks, against which computational models such as can be tested.

## Conclusions

The aim of this study was to provide publicly available norms of interpretation for common emojis as well as some code to facilitate future research in rapidly growing areas of inquiry examining how emojis are interpreted in meaningful communication, how they interact with linguistic processing, and how computers might be able to process their meaning, just to name a few. The scale response for emotional valence makes these data easily comparable to other sentiment values for emoji that already exist (e.g., Novak et al., 2015). The open-ended word associations allow for individual differences in word choice and length of response. These (multi)word associations can be analyzed with respect to subject-level factors such as participant age and sex as well as text use and emoji use. They can also be analyzed at the item-level, examining variance in emotional valence and word association for each emoji. These data can also easily be merged with other data, be they statistical properties of the words (e.g., word frequency), semantic properties of the words (e.g., concreteness ratings, semantic vectors), or properties of the emojis themselves (e.g., emoji frequency). A follow-up step in this line of research could be to improve these data by including emoji from other platforms, from older participants, and from international populations, all of which would improve our understanding of the roles of these ubiquitous features of modern communication.

## Data Availability Statement

The datasets presented in this study can be found in online repositories. The names of the repository/repositories and accession number(s) can be found in the article/Supplementary Material.

## Ethics Statement

The studies involving human participants were reviewed and approved by Kent State University Institutional Review Board. Written informed consent for participation was not required for this study in accordance with the national legislation and the institutional requirements.

## Author Contributions

CW designed and programmed the experiment, was responsible for the collection of data, and contributed to the manuscript writing and revisions. PH was the originator of the idea, organized and analyzed the data, and contributed to the writing and revisions of the manuscript. All authors contributed to the article and approved the submitted version.

## Conflict of Interest

The authors declare that the research was conducted in the absence of any commercial or financial relationships that could be construed as a potential conflict of interest.

## Footnotes

^1^We are grateful to Randall Jamieson for sharing the R code for making these vectors with us.
